# TLR4-Pathway-Associated Biomarkers in Subarachnoid Hemorrhage (SAH): Potential Targets for Future Anti-Inflammatory Therapies

**DOI:** 10.3390/ijms232012618

**Published:** 2022-10-20

**Authors:** Rebecca Heinz, Ulf C. Schneider

**Affiliations:** 1 Experimental Neurosurgery, Charité–Universitätsmedizin Berlin, Corporate Member of Freie Universität Berlin, Humboldt-Universität zu Berlin and Berlin Institute of Health, 10117 Berlin, Germany; 2 Department of Neurosurgery, Cantonal Hospital of Lucerne, 6000 Lucerne, Switzerland

**Keywords:** toll-like receptor 4, neuroinflammation, MyD88, subarachnoid hemorrhage, NLRP3 inflammasome

## Abstract

Subarachnoid hemorrhage is associated with severe neurological deficits for survivors. Among survivors of the initial bleeding, secondary brain injury leads to additional brain damage. Apart from cerebral vasospasm, secondary brain injury mainly results from cerebral inflammation taking place in the brain parenchyma after bleeding. The brain’s innate immune system is activated, which leads to disturbances in brain homeostasis, cleavage of inflammatory cytokines and, subsequently, neuronal cell death. The toll-like receptor (TLR)4 signaling pathway has been found to play an essential role in the pathophysiology of acute brain injuries such as subarachnoid hemorrhage (SAH). TLR4 is expressed on the cell surface of microglia, which are key players in the cellular immune responses of the brain. The participants in the signaling pathway, such as TLR4-pathway-like ligands, the receptor itself, and inflammatory cytokines, can act as biomarkers, serving as clues regarding the inflammatory status after SAH. Moreover, protein complexes such as the NLRP3 inflammasome or receptors such as TREM1 frame the TLR4 pathway and are indicative of inflammation. In this review, we focus on the activity of the TLR4 pathway and its contributors, which can act as biomarkers of neuroinflammation or even offer potential new treatment targets for secondary neuronal cell death after SAH.

## 1. Introduction

Subarachnoid hemorrhage (SAH) is a complex condition with high morbidity and mortality. Numerous different factors contributing to brain injury after SAH have been identified. Despite treatment of complications of early brain injury, such as rise of intracranial pressure, disturbance of the blood–brain barrier, cerebral edema, and decrease in cerebral perfusion, the outcome of many patients remains devastating. Neuroinflammation within the brain parenchyma with associated neuronal cell death has been described as a leading mechanism for additional secondary brain injury [1]. It involves complex signaling cascades in which the upregulation of inflammatory genes is induced. Consequently, the release of different inflammatory cytokines and chemokines leads to self-reinforcement of the immune system with concomitant neuronal cell death, destroying the brain. The immune system within the central nervous system (CNS) fulfills a special role. It is privileged and differs in its standards from the peripheral immune response. For example, experimentally implanted tissue grafts survive much longer when implanted into the CNS due to its immune-privileged status [2]. Therefore, neuroinflammation must be treated differently compared with inflammation in the rest of the body. In the past, the blood–brain barrier (BBB) and the lack of lymphatic vessels seemed to be responsible for this effect, whereas new investigations have shown that this is due to the presence of connections to the peripheral immune system [3]. 

Inflammation is commonly understood as the immune system reacting or responding to something it cannot recognize or remember, which can take the form of a pathogen-associated molecular pattern (PAMP) or a damage-associated molecular pattern (DAMP) resulting, for example, from dying cells [4,5]. Pattern recognition receptors (PRRs) sense these patterns and initiate an immune response. To date, five different classes of PRR have been recognized: Toll-like receptors (TLRs), which are mainly present on the cellular surface or in endosomes; NOD-like receptors (NLRs), which are located in the cytoplasm of cells; RIG-I-like receptors (RLRs), which are closely associated with viral response and are intracellularly located; C-type lectin receptors (CLRs), which are transmembrane receptors with a carbohydrate-binding domain; and absence-in-melanoma2 (AIM2)-like receptors, which are involved in the detection of intracellular microbial DNA [6,7].

One of the best characterized family of PRRs is the family of TLRs, which are mainly present on dendritic cells (DC) and macrophages. At least ten different TLRs have been identified in both humans and mice [7,8]. 

Microglia, acting as the innate immune system of the CNS, also express these receptors, reacting to the appearance of DAMPs through activation and subsequent neuroinflammation [9,10]. TLR4, in particular, has come into focus as a key player in neuroinflammation after acute neurological events [11,12,13]. Its capacity to bind specific DAMPs appearing in the diseased brain is postulated as one of the mechanisms leading to neuroinflammation.

Consequently, the TLR4 pathway is one of the most-investigated pathways regarding neuroinflammation after SAH [14,15,16]. Many ligands appear in the subarachnoid space after SAH, such as cell lysate from red blood cells (RBCs), high mobility group box 1 (HMGB1) proteins, or proangiogenic molecules such as fibrinogen. These new appearing ligands can bind to TLR4. This binding process leads to downstream signaling activity with the cleavage of proinflammatory cytokines, co-signaling of other pathways, and formation of proinflammatory inflammasomes such as NLR family pyrin domain containing 3 (NLRP3) [17,18]. 

All these components can act as biomarkers, indicating inflammatory activity, and might thus offer the potential for surveillance in addition to treatment of patients after SAH. 

Here, we review the inflammation process induced by the TLR4 pathway and describe possible biomarkers associated with this pathway along with potential treatment strategies. 

## 2. Microglia as Key Players of the CNS Innate Immune System

As the CNS is mostly independent from the peripheral immune system, it possesses its own innate immune system mainly consisting of microglia adjacent to astrocytes and oligodendrocytes. Microglia originate from the yolk sac in the embryonal stage and act as resident macrophages [19,20]. Under physiological conditions, these cells remain in a non-activated state, with a ramified morphology, and are continuously surveying their surrounding environment for potential danger. They act as the “housekeeper” of the CNS, maintaining myelin homeostasis, clearing the CNS environment of dead cells, and initiating synaptic remodeling [21,22,23]. Upon activation, they change their morphology, adopting an amoeboid appearance [24]. Reasons for activation might include the loss of signaling from injured neurons, because intercommunication between microglia and neurons is crucial for maintaining brain homeostasis in a healthy brain. Additionally, typical immune stimulators, such as DAMPs and inflammatory cytokines, can act as microglia reactivators [25]. In an activated state, microglia can cause neuroinflammation, which induces neurotoxicity [26,27]. 

Microglia can interact with other cells in the CNS that are associated with neuroinflammation. Astrocytes are reported to have a critical role in preserving neuronal function, being an essential part of the BBB but also contributing to neuroinflammation by changing their activation status to that of reactive astrocytes (RA) [28,29]. Crosstalk between these two cell types is essential for maintaining homeostasis and the development and regulation of synapsis formation in the healthy brain, but it can also have detrimental effects during neuroinflammatory events [30]. As microglia appear to be more sensitive toward PAMPs or DAMPs, they act in the front row of immune activation, especially in the context of SAH. After activation, they secrete proinflammatory cytokines, which activate astrocytes. Astrocytes do not respond to potential immune activators in such an emerging way as microglia and are, instead, predisposed towards soluble mediators such as IL1β. There is mounting evidence suggesting that astrocytes are essentially dependent on microglia in their activation manner towards different DAMPs or PAMPs [31,32]. The crosstalk between microglia and astrocytes is important, as the whole process of inflammation must be observed when considering specific inflammatory pathways and potential contributors. 

Microglia play a crucial role during inflammation after SAH. Many treatment approaches to early brain injury after SAH focus on microglia function and associated pathways, such as the TLR4 pathway, with the goal of inhibiting inflammation and its accompanying neuronal cell death [1,33,34,35].

## 3. The TLR4 Pathway

**Figure 1:**
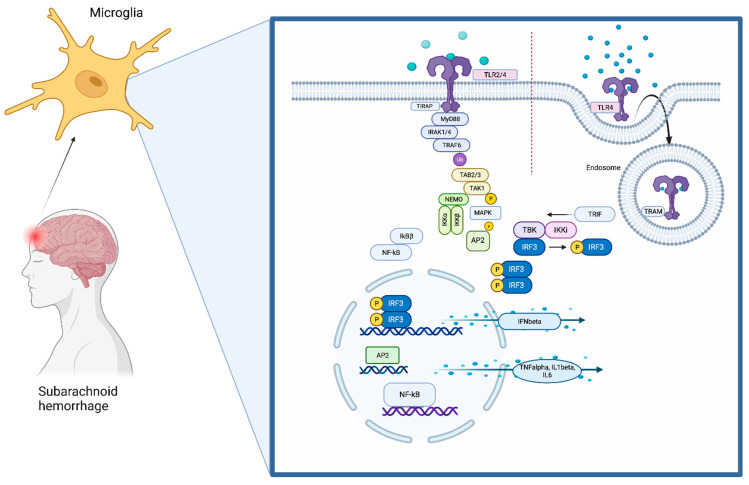
The TLR4 pathway. TLR4 is localized on the cell surface of microglia, where it senses DAMPs that are derived during brain damage. The TLR signaling pathway is activated by ligand-induced dimerization of receptors, followed by the binding of MyD88 and TIRAP or TRIF and TRAM. TLR4 translocates from the plasma membrane to endosomes with a switch from MYD88 to TRIF signaling. Activation of IRAK-1, IRAK-2, IRAK-4, and TRAFs leads to the activation of TAK1, TAK1-binding protein (TAB)1, TAB2, and TAB3. This complex phosphorylates and therefore activates IKKβ, which again forms a complex with IKKα, IKKβ, and NEMO. This leads to phosphorylation of NFκB inhibitory protein, which consequently frees NFκB. TAK1 is also involved in the phosphorylation and therefore activation of MAPKs. Transcription factors such as NFκB and AP1 induce proinflammatory cytokines. Activation of endosomal TLRs leads to the production of interferons through transcription factor IRF3. Although TLR4 has the ability to use both pathway arms, TLR2 can only signal through the MyD88-dependent pathway.

Throughout the brain, TLR4 is located on microglia (Figure 1) [35]. It recognizes its ligands together with myeloid differentiation factor 2 (MD2). The most common ligands are lipopolysaccharides (LPS), which are structures derived from the outer membrane of Gram-negative bacteria. When binding to a ligand, two TLR4–MD2 complexes, together with the ligand, are required to build a TLR4 homodimer [36].

TLR4 contains N-terminal leucine-rich repeats (LRRs) and a transmembrane region, which is followed by a Toll/IL1R homology (TIR) domain. The TIR domain is connected to adaptor molecules (TIRAP). TLR4 signaling involves mainly myeloid differentiation primary response protein 88 (MyD88) and TIR-domain-containing adaptor-inducing IFN-beta (TRIF). The pathway then divides into the MyD88-dependent or the MyD88-independent pathway depending on which TIR domain is used [6,37,38,39]. 

Taken together, activation of TLR4 mainly triggers inflammation through production of proinflammatory cytokines by releasing the transcription factors NFκB and AP1 into the nucleus. The mechanisms balancing the production of anti-inflammatory type-1 interferons over TRIF-mediated TLR4 signaling and the production of proinflammatory cytokines through activation of NFκB and MAPKs are not fully understood yet. Signaling protein TRAF3 might play a decisive role in determining in which direction the pathway proceeds [40,41] Huai and colleagues reported that the tyrosine phosphatase PTPN4 dephosphorylates and, as a result, inhibits tyrosine phosphorylation of TRAM and thereby specifically inhibits the TRIF-dependent pathway [42].

## 4. Crosstalk of TLR4 and the NLRP3 Inflammasome

When analyzing inflammatory processes associated with SAH, the contribution of the inflammasome seems inevitable [43]. Inflammasomes are cytosolic multiprotein complexes that can activate proinflammatory caspase-1 through autoproteolytic activation of procaspase-1. As a consequence, this leads to the cleavage of pro-inflammatory cytokines, mainly IL1β and IL18, initiating an inflammatory immune response [44]. Caspase-1 also induces an inflammatory form of cell death named pyroptosis, which is highly associated with inflammasome activation [45].

Currently, the best characterized inflammasome is the NLRP3 inflammasome, which consists of the NLRP3 scaffold, a sensor, an adaptor domain (ASC), and procaspase-1, the effector. ASC consists of a C-terminal caspase activation and recruitment (CARD) domain and an N-terminal pyrin domain (PYD), which enables bridging of the sensor and the effector [44]. In the CNS, inflammasomes are mainly present in microglia but have also been detected in neurons [46]. Similarly to the TLR4 pathway, the NLRP3 inflammasome can become activated not only through pathogens but also host-derived DAMPs arising from tissue damage resulting from sterile injury as seen during SAH [47].

However, the activation and signaling of inflammasomes is a complex process, where many different activators can potentially contribute.

To activate an inflammasome, a two-step procedure is necessary, which requires two different signals. The priming step leads to the transduction and translation of inflammasome components (NLRP3, caspase-1, ASC, proIL1β). This first signal often appears through signaling using PRR, such as TLR4, which activates NFκB to induce transcription of NLRP3 and pro-IL1β. 

The second signal, the activation step, triggers the formation of the inflammasome complex. A wide range of stimuli in the cytosol have been found to initiate the activation of NLRP3, including potassium efflux, calcium accumulation, ATP, and cathepsin release through lysosomal damage or mitochondrial-injury-induced ROS production, to name but a few of the most investigated mechanisms [46,48]. 

**Figure 2:**
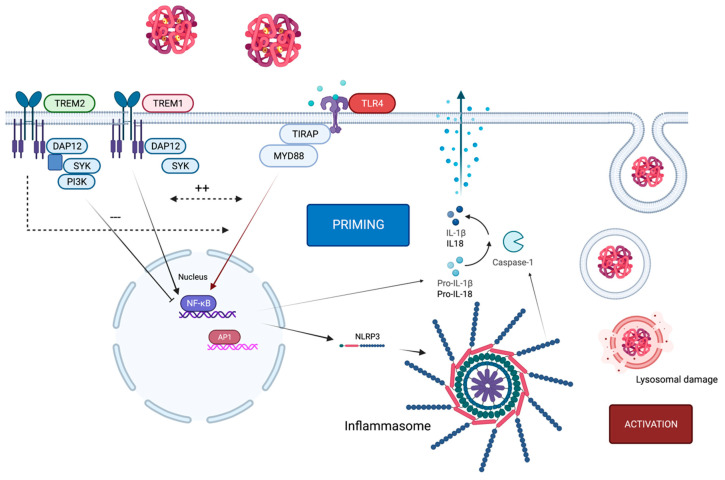
TLR4 in context of TREM1/2 and the NLRP3 inflammasome. Activation of TLR4 through hemoglobin causes expression of the NLRP3 inflammasome, which initiates the priming step. For activation, lysosomal damage (as one example of many potential activation mechanisms) induces activation of the NLRP3 inflammasome. Upon ligand binding, the TREM2-associated adaptor DAP12 undergoes tyrosine phosphorylation by the protein kinase SRC and recruits the tyrosine protein kinase SYK, which activates phosphatidylinositol 3-kinase. TREM2 limits inflammation by interfering with proinflammatory signals, which can be transmitted by TLR4. By contrast, the TREM1 signaling cascade reinforces the production of inflammatory mediators that are initiated by TLR4 and act synergistically. TREM1 acts here as an amplifier of TLR-induced inflammation and vice versa.

TLR4 activation through DAMPs appears to be the priming signal for inflammasome activation via activation of NFκB and therefore transcription of NLRP3 and pro-IL1β. DAMPs such as in red blood cell lysate or HMGB1 can act as signal 1 through binding to TLR4 but also as a secondary signal by binding to the inflammasome when phagocytosed by microglia [49,50,51]. This leads to self-reinforcement of TLR4 and NLRP3 with exacerbation of the inflammatory response [52,53] (Figure 2). 

Interestingly, IFNβ, also a product of the TLR4 pathway, is reported to have an immunomodulatory effect on the NLRP3 inflammasome, as it is able to suppress caspase-1 activity and therefore IL1β-expression [54]. 

Thus, the inflammasome is dependent on TLR4. This might be a double-edged sword, as the inflammatory part of the TLR4 pathway can induce NLRP3 activation, but the immunomodulatory part, leading through IRF3 to IFNβ expression, can inhibit IL1β release.

Particularly in SAH, the NLRP3 inflammasome is reported to be part of the innate immune response appearing after the event [17,55,56].

## 5. Co-Existence of TLR4 Alongside TREM1/2

Alongside TLR4, other receptors and pathways exist that have been reported to influence the immune response in the brain. 

The triggering receptor expressed on myeloid cells (TREM) is a relatively new player in the field of cell surface receptors. Discovered in 2000 [57], the family increased from only one (TREM-1) to three receptors, all of which have slightly different tasks. Here, we focus on TREM1 and TREM2 as they have both been reported to be present on microglial surfaces. TREM1 and TREM2 are better known for their contribution in Alzheimer disease, but in the last couple of years, many investigations have been conducted regarding their contribution to inflammatory processes after SAH [47]. 

TREM1 is an amplifier of inflammatory signaling and acts synergistically with TLRs and NLRs in the production of inflammatory cytokines. With its discovery, it was identified on neutrophils and monocytes [57]. However, the signaling pathway differs from that of the TLR4 pathway, containing the adaptor protein DAP12, though it is also involved in NFκB activation. Initially, only PAMPs were identified as ligands of TREM1, indicating that it might have a crucial role in microbial contexts such as sepsis [58]. More recently, the role of TREM1 in neuroinflammatory diseases was also investigated, and the presence of TREM1 on microglia was identified [59]. In Alzheimer disease (AD), TREM1 was found to be important for the phagocytosis ability of microglia and therefore lowered the amyloid-β burden in the brain when overexpressed [60,61], having a beneficial effect. In contrast, TREM1 was reported to be crucial for the inflammatory response after SAH (Figure 2). Blocking TREM1 reduced neuronal cell death and ameliorated inflammatory cell recruitment in the brain after SAH [47,62].

However, additional evidence suggests TREM2 has a positive impact on the neuroinflammatory response after acute brain injuries such as intracerebral hemorrhage or SAH, acting contrary to TREM1. Triggering TREM2 activity with apoE, a strong ligand of TREM2, is associated with reduced cerebral edema and inhibited microglial activity after SAH [63]. 

TREM activity is associated with TLR signaling [64,65]. Hamerman and colleagues investigated an increase in TLR response in DAP12-deficient mice (for which TREM signaling is therefore not possible). These findings suggest the existence of a synergic effect between TLRs and TREMs [66], hinting that the immune system allows crosstalk between different pathways and receptors and could compensate for the failure of a single pathway or receptor. Moreover, an imbalance in the TREM2/TLR4 ratio has been reported to have detrimental effects regarding neuroinflammation in Alzheimer Disease [67]. 

## 6. Pathophysiology of Inflammation after SAH

Among survivors of the initial bleeding after SAH, secondary brain injury caused by neuroinflammation is the reason for major morbidity [68,69]. In the past decades, cerebral vasospasm was the most investigated mechanism associated with secondary brain injury. However, clinical trials applying anti-vasospasm drugs could not show a beneficial effect in terms of clinical outcome [70]. Thus, recent evidence suggests a major role for neuroinflammation after SAH as a key contributor to injury expansion and cognitive deficits in the context of secondary brain injury after SAH [71,72,73]. 

The appearance of blood after SAH in the subarachnoid space not only raises ICP and leads to general ischemia but also acts as a potential immune stimulus. The lysis of erythrocytes leads to the deposition of hemoglobin [18,74]. As already discussed above, hemoglobin derivates stimulate TLR4 as DAMPs. As a consequence, microglia are activated after SAH. They fulfill their role as edaphic macrophages with phagocytosis but also with cleavage of inflammatory molecules [1,75]. 

Our group has described an outside-in activation with intravascular inflammation followed by activation of microglia at the vessel/brain interface [76]. It is also known that the blood–brain barrier (BBB) is severely damaged after SAH, with disrupted tight junctions, transcytosis, and endothelial dysfunction [77], implying that the damaged BBB is more permeable to potential immune-stimulating molecules. Blecharz-Lang and colleagues postulated that the proinflammatory cytokine IL6 might be responsible for BBB disruption [78]. 

The TLR4 downstream pathway ultimately leads to the cleavage of proinflammatory cytokines such as IL1β and IL6 [79]. Other inflammatory cells, such as neutrophils and macrophages, are recruited upon microglia activation into the subarachnoid space through upregulation of intercellular adhesion molecule 1 (ICAM1), where they phagocytose any remaining erythrocyte breakdown products [76,80]. Conversely, it is also reported that neutrophil recruitment along the vessels trigger microglia activation, which leads to a vicious circle of self-reinforcing inflammation [76]. It was recently reported that meningeal lymphatics are also involved in clearing extravasated erythrocytes after SAH [81]. The immune cells become imprisoned in the subarachnoid space, where they release vasoactive factors such as endothelins, which may contribute to vessel reactions such as vasospasm [82]. However, the connection between cerebral inflammation and cerebral vasospasm after SAH is still not fully understood.

Analysis of the amount of proinflammatory cytokines in the cerebrospinal fluid (CSF) and serum of SAH patients showed the correlation of high IL6 and TNFα levels with poor functional outcome [83,84], indicating not only an inflammation state restricted to the brain but spreading systemically and affecting the whole immune system.

Recently, it was reported that TLR4 signaling is also involved in the risk of aneurysmal rupture by triggering vascular inflammation. TLR4 is expressed on endothelial cells, which may be an explanation for the involvement of vessel inflammation as a result of TLR4 activation. TLR4/MyD88 KO mice had a reduction in aneurysm rupture rates but not in incidence of aneurysm formation [85,86,87]. 

## 7. TLR4-Pathway-Associated Proteins Acting as Biomarkers following SAH 

### 7.1. Ligands of TLR4

DAMPs are physiologically sequestered intracellularly and are therefore invisible to the immune system. If cells die or become damaged due to neuronal damage after SAH, these patterns then become visible to immune cells of the CNS, mainly for microglia and their receptors [7]. Even though the list of potential DAMPs is growing constantly and is becoming increasingly heterogenous, their presence homogenously results in inflammation with expression of inflammation-related molecules and cytokines, iNOS, or COX2. Therefore, they are closely linked with neuroinflammatory activity after SAH and can work as biomarkers in predicting disease progression and potential anti-inflammatory therapeutic outcome [88].

#### 7.1.1. HMGB1

HMGB1 is a DNA-binding protein which is located in the nucleus of neurons. When secreted extracellularly through active release or cell death, it interacts with its main receptors TLR4 and the receptor for advanced glycation end products (RAGE) as a DAMP [89]. 

It was shown that HMGB1 levels in the CSF and plasma of patients suffering from SAH are positively correlated not only with IL6 and TNFα levels but also with the severity and the clinical outcome [50,90,91,92]. 

Reduction in mRNA and protein expression of HMGB1 upon treatment with the natural HMGB1 inhibitor glycyrrhizin was shown to improve neurological scores in an experimental model of SAH [93]. 

HMGB1 mAB were also used in preclinical studies focusing on early brain injury after SAH, where a significant decrease in inflammatory cytokines and associated brain injury could be detected. Furthermore, delayed cerebral vasospasm was attenuated [94]. This observation aligns with the findings of Nakahara and colleagues, showing that high CSF and plasma levels of HMGB1 are correlated with poor neurological outcome after SAH [50]. Zhu and colleagues showed that HMGB1 is predictive of poor functional outcome after 1 year, but not for the prediction of mortality after 1 year, in-hospital mortality, or vasospasm [92]. 

#### 7.1.2. Red Blood Cell Lysate 

During subarachnoid hemorrhage, the brain is exposed to blood particles and cell lysates of erythrocytes. Therefore, hemoglobin and its constituents moved into focus regarding initiation of an immune response by binding to TLR4 on the surface of microglia [18,95].

Wang and colleagues measured the levels of heme oxygenase-1 (HO-1), oxyhemoglobin, ferritin, and bilirubin in intrathecal CSF on the 7th day post-hemorrhage. HO-1 appeared to be the most significant CSF parameter related to an unfavorable outcome [96]. 

In contrast, Frase and colleagues also described increased CSF HO-1 levels in patients after SAH, but with no significant difference in functional outcome. However, lower HO-1 levels on day 7 post-SAH were associated with vasospasm [97]. 

Oxyhemoglobin (OxyHb) is released during the lysis of red blood cells and interacts as a DAMP with TLR4 [34]. It autoxidizes to methemoglobin, which is also shown to be a TLR4 ligand [49], as well as heme, another hemolysis-associated DAMP [98,99]. Heme metabolism results in the breakdown products free iron and bilirubin. Free iron, in particular, is postulated to catalyze the formation of reactive oxygen species (ROS), which act as a strong inflammatory activator by binding to TLR4 [99]. 

### 7.2. Inflammatory Cytokines 

Particularly in the CNS, where the initial immune response is triggered by microglia and in part by astrocytes, inflammatory cytokines play an emerging role when it comes to spreading inflammation in the brain [100].

Interleukin (IL)-6 seems to be an important interleukin in the inflammatory response after SAH, and many studies describe a strong correlation between elevated IL6 levels in plasma and CSF and worse outcomes [83,101,102]. 

TNFα was also described as being elevated in CSF and plasma in SAH compared with healthy controls, and higher CSF levels are linked with poorer outcomes [103,104].

In clinical phase II studies, significantly lower levels of IL6 and CRP as inflammatory markers were observed in SAH patients after subcutaneous administration of IL1RA compared to a placebo group. However, the clinical outcome was not improved significantly [105,106]. CSF-IL6, but not IL6, plasma levels are significantly correlated with the development of delayed brain injury after SAH, indicating that inflammation-triggered brain injury is a relevant problem not just in theory or preclinical trials but also in clinical settings [107]. Analogous to the SAH studies, IL1RA was also applied subcutaneously to patients after suffering from cerebral ischemia, where diminished IL6 plasma concentrations and a better clinical outcome were observed in the treated group [108].

### 7.3. TLR4 

TLR4 itself was also investigated in SAH patients to be a potential biomarker. SAH patients were found to have higher TLR4 expression levels on the cell surface of peripheral blood mononuclear cells. TLR4 expression was determined by flow cytometry, and patients were assessed every day after admission to monitor the occurrence of delayed cerebral ischemia (DCI). Patients with DCI showed significantly higher TLR4 levels than those without DCI [14]. Another study demonstrated increased levels of TLR4 in the CSF of patients 3 days after SAH [109]. 

TAK-242 binds to the intracellular domain of TLR4 and selectively inhibits TLR4 signaling [110]. It has been shown to ameliorate brain damage caused by neuroinflammation in SAH [111,112].

Another drug which has been reported to downregulate TLR4 expression is fluoxetine. Fluoxetine is a common selective serotonin reuptake inhibitor (SSRI) that also has the ability to downregulate TLR4 and the NLRP3 inflammasome after SAH [113]. However, the exact mechanisms behind the neuroprotective effects of fluoxetine remain elusive. 

Polyphenols have also been described as modulators of the TLR4 pathway. They include a wide variety of molecules which all share a similar basic polyphenolic structure. They serve to protect against a variety of pathogens with anti-inflammatory and antioxidant properties [114]. Resveratrol, a potential neuroprotective polyphenol, has the ability to inhibit murine RAW 264.7 macrophages und microglial BV-2 cells, and it also inhibits downstream phosphorylation of STAT1 und STAT3. Flavonoid derivates were shown to inhibit LPS-induced TLR4 dimerization in RAW 264.7 macrophage lines. Luteolin, another polyphenol, suppresses activation of IRF3 and NFκB induced by TLR4 agonists with similar effects as flavonoids or resveratrol: attenuation of inflammatory cytokines, cleaved through activation of the TRIF-dependent pathway [115,116,117]. Curcumin, a polyphenolic compound, has been shown to suppress TLR4-positive microglia in the brain and therefore lead to diminished LPS-induced IRF3 activation [118].

However, therapeutical approaches directly targeting TLR4 have not been performed in clinical studies but only in experimental preclinical settings. 

### 7.4. TREM1 

TREM1 is closely correlated with TLR4 pathway activation (as mentioned above). It has been investigated as one of the amplifiers of various inflammatory diseases. Concentrations of soluble TREM1 (sTREM1) have been assessed in the CSF of SAH patients. Sun and colleagues explored the correlations of early CSF sTREM1 levels with severity and prognosis and found that sTREM1 levels in CSF of SAH patients were significantly increased compared with healthy controls, and correlated negatively with Glasgow Coma Scale (GCS) and positively with the Hunt and Hess scale [119]. The same group stated that inhibition of TREM1 with LP17, a synthetic TREM1 inhibitor, ameliorated microglial pyroptosis and IL1β production in experimental SAH. They also found that TREM1 can activate the NLRP3 inflammasome, which is also closely correlated with the TLR4 pathway [47]. 

### 7.5. Inflammasome Proteins 

The NLRP3 inflammasome plays an important role in the inflammatory process in many diseases affecting the brain [46]. As its activation is closely associated with the TLR4 pathway, it should also be considered when discussing potential biomarkers and treatment strategies focusing on neuroinflammation after SAH. 

Caspase-1 is a product of the inflammatory cascade found after inflammasome activation, and its occurrence in CSF was tested in 18 SAH patients. CSF analysis demonstrated a nearly seven-fold increase in caspase-1 activity in SAH patients compared with controls. Mean caspase-1 activity in the poor outcome group was approximately three times higher than the good outcome group and, furthermore, caspase-1 activity was significantly correlated with GOS score and clinical outcome [120]. 

Another study not only assessed caspase-1 but also inflammasome proteins such as ASC and NLRP1. They also found that higher levels of inflammasome proteins were associated with severe SAH and poor outcome 3 months after SAH [121].

MCC950 is a small-molecule inhibitor of the NLRP3 inflammasome that interacts with the NACHT domain and inhibits ATP hydrolysis [122]. It has been found to have beneficial effects in ischemic stroke, where it leads to decreased TNFα levels, improved neurological outcome, reduced cerebral edema, and decreased infarction size [123,124] in animal studies. 

## 8. Conclusions

Neuroinflammation has become one of the most investigated fields regarding poor neurological outcome after several acute neurological disorders such as SAH. 

The TLR4 pathway is prominent in many neurological disorders associated with neuroinflammation. DAMPs, appearing in the CNS due to various dysregulations or injuries, bind to the TLR4, leading to downstream signaling that culminates in the release of proinflammatory molecules. Of course, the TLR4 pathway acts not reclusively without any co-contributors but is framed by other pathways and molecular protein complexes such as the NLRP3 inflammasome. 

Different components of the TLR4 pathway can act as biomarkers for indicating the inflammatory status, not only of the brain but also systemically. 

Focused therapy attempts targeting these components with antagonizing antibodies or small-molecule inhibitors have already shown promise in past studies and might further elicit remarkable effects in future studies. 

Neuroinflammation, with special regard to the TLR4 pathway, has increasingly come into focus in discussions of individualized therapies after SAH to protect survivors from unfavorable outcomes. Therefore, the abovementioned contributors of this pathway can be useful targets in clinical settings to obtain information about the inflammatory status and might furthermore provide potential targets for future anti-inflammatory therapies. 

## Data Availability

Not applicable.

## Abbreviations

SAH	subarachnoid hemorrhage
TLR4	Toll-like receptor 4
CNS	central nervous system
BBB	blood–brain barrier
DAMP	danger-associated molecular pattern
PAMP	pathogen-associated molecular pattern
PRR	pattern recognition receptor
MyD88	myeloid differentiation primary response protein 88 (MyD88)
TRIF	TIR-domain-containing adaptor-inducing IFN-beta
MAPK	mitogen-activated protein kinase
TREM	triggering receptor expressed on myeloid cells
HMGB1	high mobility group box 1
NLRP3	NLR family pyrin domain containing 3
ICAM1	intercellular adhesion molecule 1
ROS	reactive oxygen species
COX 2	Cyclooxygenase 2
TREM	triggering receptor expressed on myeloid cells

## References

[1] Schneider U.C., Davids A.M., Brandenburg S., Muller A., Elke A., Magrini S., Atangana E., Turkowski K., Finger T., Gutenberg A. (2015). Microglia inflict delayed brain injury after subarachnoid hemorrhage. Acta Neuropathol..

[2] Medawar P.B. (1948). Immunity to homologous grafted skin; the fate of skin homografts transplanted to the brain, to subcutaneous tissue, and to the anterior chamber of the eye. Br. J. Exp. Pathol..

[3] Engelhardt B., Vajkoczy P., Weller R.O. (2017). The movers and shapers in immune privilege of the CNS. Nat. Immunol..

[4] Kono H., Rock K.L. (2008). How dying cells alert the immune system to danger. Nat. Rev. Immunol..

[5] Medzhitov R., Janeway C.A. (2002). Decoding the patterns of self and nonself by the innate immune system. Science.

[6] Takeuchi O., Akira S. (2010). Pattern recognition receptors and inflammation. Cell.

[7] Chen G.Y., Nunez G. (2010). Sterile inflammation: Sensing and reacting to damage. Nat. Rev. Immunol..

[8] Tsan M.F., Gao B. (2004). Endogenous ligands of Toll-like receptors. J. Leukoc. Biol..

[9] Pineau I., Lacroix S. (2009). Endogenous signals initiating inflammation in the injured nervous system. Glia.

[10] Balanca B., Desmurs L., Grelier J., Perret-Liaudet A., Lukaszewicz A.C. (2021). DAMPs and RAGE Pathophysiology at the Acute Phase of Brain Injury: An Overview. Int. J. Mol. Sci..

[11] Li Y., Zhang L., Tang J., Yang X., Huang J., Zhu T., Zhao F., Li S., Li X., Qu Y. (2019). Role of toll-like receptor 4 in the regulation of the cell death pathway and neuroinflammation. Brain Res. Bull.

[12] Buchanan M.M., Hutchinson M., Watkins L.R., Yin H. (2010). Toll-like receptor 4 in CNS pathologies. J. Neurochem..

[13] Li L., Acioglu C., Heary R.F., Elkabes S. (2021). Role of astroglial toll-like receptors (TLRs) in central nervous system infections, injury and neurodegenerative diseases. Brain Behav. Immun..

[14] Ma C., Zhou W., Yan Z., Qu M., Bu X. (2015). Toll-like receptor 4 (TLR4) is correlated with delayed cerebral ischemia (DCI) and poor prognosis in aneurysmal subarachnoid hemorrhage. J. Neurol. Sci..

[15] Okada T., Suzuki H. (2017). Toll-like receptor 4 as a possible therapeutic target for delayed brain injuries after aneurysmal subarachnoid hemorrhage. Neural Regen. Res..

[16] Weiland J., Beez A., Westermaier T., Kunze E., Siren A.L., Lilla N. (2021). Neuroprotective Strategies in Aneurysmal Subarachnoid Hemorrhage (aSAH). Int. J. Mol. Sci..

[17] Zhang X., Wu Q., Zhang Q., Lu Y., Liu J., Li W., Lv S., Zhou M., Zhang X., Hang C. (2017). Resveratrol Attenuates Early Brain Injury after Experimental Subarachnoid Hemorrhage via Inhibition of NLRP3 Inflammasome Activation. Front. Neurosci..

[18] Chaudhry S.R., Hafez A., Jahromi B.R., Kinfe T.M., Lamprecht A., Niemela M., Muhammad S. (2018). Role of Damage Associated Molecular Pattern Molecules (DAMPs) in Aneurysmal Subarachnoid Hemorrhage (aSAH). Int. J. Mol. Sci..

[19] Kettenmann H., Hanisch U.K., Noda M., Verkhratsky A. (2011). Physiology of microglia. Physiol. Rev..

[20] Ginhoux F., Greter M., Leboeuf M., Nandi S., See P., Gokhan S., Mehler M.F., Conway S.J., Ng L.G., Stanley E.R. (2010). Fate mapping analysis reveals that adult microglia derive from primitive macrophages. Science.

[21] Hickman S., Izzy S., Sen P., Morsett L., EI Khoury J. (2018). Microglia in neurodegeneration. Nat. Neurosci..

[22] Vasek M.J., Garber C., Dorsey D., Durrant D.M., Bollman B., Soung A., Yu J., Perez-Torres C., Frouin A., Wilton D.K. (2016). A complement-microglial axis drives synapse loss during virus-induced memory impairment. Nature.

[23] Lui H., Zhang J., Makinson S.R., Cahill M.K., Kelley K.W., Huang H.Y., Shang Y., Oldham M.C., Martens L.H., Gao F. (2016). Progranulin Deficiency Promotes Circuit-Specific Synaptic Pruning by Microglia via Complement Activation. Cell.

[24] Wolf S.A., Boddeke H.W., Kettenmann H. (2017). Microglia in Physiology and Disease. Annu. Rev. Physiol..

[25] Ransohoff R.M. (2016). A polarizing question: Do M1 and M2 microglia exist?. Nat. Neurosci..

[26] Deczkowska A., Keren-Shaul H., Weiner A., Colonna M., Schwartz M., Amit I. (2018). Disease-Associated Microglia: A Universal Immune Sensor of Neurodegeneration. Cell.

[27] Song W.M., Colonna M. (2018). The identity and function of microglia in neurodegeneration. Nat. Immunol..

[28] Orihuela R., McPherson C.A., Harry G.J. (2016). Microglial M1/M2 polarization and metabolic states. Br. J. Pharmacol..

[29] Almad A., Maragakis N.J. (2018). A stocked toolbox for understanding the role of astrocytes in disease. Nat. Rev. Neurol..

[30] Liu L.R., Liu J.C., Bao J.S., Bai Q.Q., Wang G.Q. (2020). Interaction of Microglia and Astrocytes in the Neurovascular Unit. Front. Immunol..

[31] Liddelow S.A., Guttenplan K.A., Clarke L.E., Bennett F.C., Bohlen C.J., Schirmer L., Bennett M.L., Munch A.E., Chung W.S., Peterson T.C. (2017). Neurotoxic reactive astrocytes are induced by activated microglia. Nature.

[32] Liddelow S.A., Barres B.A. (2017). Reactive Astrocytes: Production, Function, and Therapeutic Potential. Immunity.

[33] Heinz R., Brandenburg S., Nieminen-Kelha M., Kremenetskaia I., Boehm-Sturm P., Vajkoczy P., Schneider C.U. (2021). Microglia as target for anti-inflammatory approaches to prevent secondary brain injury after subarachnoid hemorrhage (SAH). J. Neuroinflammation.

[34] Khey K.M.W., Huard A., Mahmoud S.H. (2020). Inflammatory Pathways Following Subarachnoid Hemorrhage. Cell Mol. Neurobiol..

[35] Hanafy K.A. (2013). The role of microglia and the TLR4 pathway in neuronal apoptosis and vasospasm after subarachnoid hemorrhage. J. Neuroinflammation.

[36] Park B.S., Song D.H., Kim H.M., Choi B.S., Lee H., Lee J.O. (2009). The structural basis of lipopolysaccharide recognition by the TLR4-MD-2 complex. Nature.

[37] Yamamoto M., Sato S., Hemmi H., Hoshino K., Kaisho T., Sanjo H., Takeuchi O., Sugiyama M., Okabe M., Takeda K. (2003). Role of adaptor TRIF in the MyD88-independent toll-like receptor signaling pathway. Science.

[38] Akira S., Uematsu S., Takeuchi O. (2006). Pathogen recognition and innate immunity. Cell.

[39] Palsson-McDermott E.M., Doyle S.L., McGettrick A.F., Hardy M., Husebye H., Banahan K., Gong M., Golenbock D., Espevik T., O’Neill L.A. (2009). TAG, a splice variant of the adaptor TRAM, negatively regulates the adaptor MyD88-independent TLR4 pathway. Nat. Immunol..

[40] Tseng P.H., Matsuzawa A., Zhang W., Mino T., Vignali D.A., Karin M. (2010). Different modes of ubiquitination of the adaptor TRAF3 selectively activate the expression of type I interferons and proinflammatory cytokines. Nat. Immunol..

[41] Hoebe K., Du X., Georgel P., Janssen E., Tabeta K., Kim S.O., Goode J., Lin P., Mann N., Mudd S. (2003). Identification of Lps2 as a key transducer of MyD88-independent TIR signalling. Nature.

[42] Huai W., Song H., Wang L., Li B., Zhao J., Han L., Gao C., Jiang G., Zhang L., Zhao W. (2015). Phosphatase PTPN4 preferentially inhibits TRIF-dependent TLR4 pathway by dephosphorylating TRAM. J. Immunol..

[43] Liu Y., Dai Y., Li Q., Chen C., Chen H., Song Y., Hua F., Zhang Z. (2020). Beta-amyloid activates NLRP3 inflammasome via TLR4 in mouse microglia. Neurosci. Lett..

[44] Schroder K., Tschopp J. (2010). The inflammasomes. Cell.

[45] Rathinam V.A., Vanaja S.K., Fitzgerald K.A. (2012). Regulation of inflammasome signaling. Nat. Immunol..

[46] Walsh J.G., Muruve D.A., Power C. (2014). Inflammasomes in the CNS. Nat. Rev. Neurosci..

[47] Xu P., Hong Y., Xie Y., Yuan K., Li J., Sun R., Zhang X., Shi X., Li R., Wu J. (2020). TREM-1 Exacerbates Neuroinflammatory Injury via NLRP3 Inflammasome-Mediated Pyroptosis in Experimental Subarachnoid Hemorrhage. Transl. Stroke Res..

[48] He Y., Hara H., Nunez G. (2016). Mechanism and Regulation of NLRP3 Inflammasome Activation. Trends Biochem. Sci..

[49] Kwon M.S., Woo S.K., Kurland D.B., Yoon S.H., Palmer A.F., Banerjee U., Iqbal S., Ivanova S., Gerzanich V., Simard J.M. (2015). Methemoglobin is an endogenous toll-like receptor 4 ligand-relevance to subarachnoid hemorrhage. Int. J. Mol. Sci..

[50] Nakahara T., Tsuruta R., Kaneko T., Yamashita S., Fujita M., Kasaoka S., Hashiguchi T., Suzuki M., Maruyama I., Maekawa T. (2009). High-mobility group box 1 protein in CSF of patients with subarachnoid hemorrhage. Neurocrit. Care.

[51] Zhong W.J., Duan J.X., Liu T., Yang H.H., Guan X.X., Zhang C.Y., Yang J.T., Xiong J.B., Zhou Y., Guan C.X. (2020). Activation of NLRP3 inflammasome up-regulates TREM-1 expression in murine macrophages via HMGB1 and IL-18. Int. Immunopharmacol..

[52] Yang J., Wise L., Fukuchi K.I. (2020). TLR4 Cross-Talk With NLRP3 Inflammasome and Complement Signaling Pathways in Alzheimer’s Disease. Front. Immunol..

[53] Ising C., Venegas C., Zhang S., Scheiblich H., Schmidt S.V., Vieira-Saecker A., Schwartz S., Albasset S., McManus R.M., Tejera D. (2019). NLRP3 inflammasome activation drives tau pathology. Nature.

[54] Guarda G., Braun M., Staehli F., Tardivel A., Mattmann C., Forster I., Farlik M., Decker T., Pasquier R.A.D., Romero P. (2011). Type I interferon inhibits interleukin-1 production and inflammasome activation. Immunity.

[55] Yang S.J., Shao G.F., Chen J.L., Gong J. (2018). The NLRP3 Inflammasome: An Important Driver of Neuroinflammation in Hemorrhagic Stroke. Cell Mol. Neurobiol..

[56] Gao L., Dong Q., Song Z., Shen F., Shi J., Li Y. (2017). NLRP3 inflammasome: A promising target in ischemic stroke. Inflamm. Res..

[57] Bouchon A., Dietrich J., Colonna M. (2000). Cutting edge: Inflammatory responses can be triggered by TREM-1, a novel receptor expressed on neutrophils and monocytes. J. Immunol..

[58] Nathan C., Ding A. (2001). TREM-1: A new regulator of innate immunity in sepsis syndrome. Nat. Med..

[59] Ford J.W., McVicar D.W. (2009). TREM and TREM-like receptors in inflammation and disease. Curr. Opin. Immunol..

[60] Jiang T., Zhang Y.D., Gao Q., Zhou J.S., Zhu X.C., Lu H., Shi J.Q., Tan L., Chen Q., Yu J.T. (2016). TREM1 facilitates microglial phagocytosis of amyloid beta. Acta Neuropathol..

[61] Replogle J.M., Chan G., White C.C., Raj T., Winn P.A., Evans D.A., Sperling R.A., Chibnik L.B., Bradshaw E.M., Schneider J.A. (2015). A TREM1 variant alters the accumulation of Alzheimer-related amyloid pathology. Ann. Neurol..

[62] Xu P., Zhang X., Liu Q., Xie Y., Shi X., Chen J., Li Y., Guo H., Sun R., Hong Y. (2019). Microglial TREM-1 receptor mediates neuroinflammatory injury via interaction with SYK in experimental ischemic stroke. Cell Death Dis..

[63] Chen S., Peng J., Sherchan P., Ma Y., Xiang S., Yan F., Zhao H., Jiang Y., Wang N., Zhang J.H. (2020). TREM2 activation attenuates neuroinflammation and neuronal apoptosis via PI3K/Akt pathway after intracerebral hemorrhage in mice. J. Neuroinflammation.

[64] Guerreiro R., Wojtas A., Bras J., Carrasquillo M., Rogaeva E., Majounie E., Cruchaga C., Sassi C., Kauwe J.S., Younkin S. (2013). TREM2 variants in Alzheimer’s disease. N. Engl. J. Med..

[65] Colonna M., Wang Y. (2016). TREM2 variants: New keys to decipher Alzheimer disease pathogenesis. Nat. Rev. Neurosci..

[66] Hamerman J.A., Tchao N.K., Lowell C.A., Lanier L.L. (2005). Enhanced Toll-like receptor responses in the absence of signaling adaptor DAP12. Nat. Immunol..

[67] Long H., Zhong G., Wang C., Zhang J., Zhang Y., Luo J., Shi S. (2019). TREM2 Attenuates Abeta1-42-Mediated Neuroinflammation in BV-2 Cells by Downregulating TLR Signaling. Neurochem. Res..

[68] Macdonald R.L., Schweizer T.A. (2017). Spontaneous subarachnoid haemorrhage. Lancet.

[69] Cahill J., Calvert J.W., Zhang J.H. (2006). Mechanisms of early brain injury after subarachnoid hemorrhage. J. Cereb. Blood Flow Metab..

[70] Macdonald R.L., Higashida R.T., Keller E., Mayer S.A., Molyneux A., Raabe A., Vajkoczy P., Wanke I., Bach D., Frey A. (2011). Clazosentan, an endothelin receptor antagonist, in patients with aneurysmal subarachnoid haemorrhage undergoing surgical clipping: A randomised, double-blind, placebo-controlled phase 3 trial (CONSCIOUS-2). Lancet Neurol..

[71] Cahill J., Zhang J.H. (2009). Subarachnoid hemorrhage: Is it time for a new direction?. Stroke.

[72] Suzuki H. (2019). Inflammation: A Good Research Target to Improve Outcomes of Poor-Grade Subarachnoid Hemorrhage. Transl. Stroke Res..

[73] De Oliveira Manoel A.L., Macdonald R.L. (2018). Neuroinflammation as a Target for Intervention in Subarachnoid Hemorrhage. Front. Neurol..

[74] Lucke-Wold B.P., Logsdon A.F., Manoranjan B., Turner R.C., McConnell E., Vates G.E., Huber J.D., Rosen C.L., Simard J.M. (2016). Aneurysmal Subarachnoid Hemorrhage and Neuroinflammation: A Comprehensive Review. Int. J. Mol. Sci..

[75] Gris T., Laplante P., Thebault P., Cayrol R., Najjar A., Joannette-Pilon B., Brillant-Marquis F., Magro E., English S.W., Lapointe R. (2019). Innate immunity activation in the early brain injury period following subarachnoid hemorrhage. J. Neuroinflammation.

[76] Atangana E., Schneider U.C., Blecharz K., Magrini S., Wagner J., Nieminen-Kelha M., Kremenetskaia I., Heppner F.L., Engelhardt B., Vajkoczy P. (2017). Intravascular Inflammation Triggers Intracerebral Activated Microglia and Contributes to Secondary Brain Injury After Experimental Subarachnoid Hemorrhage (eSAH). Transl. Stroke Res..

[77] Fang Y., Gao S., Wang X., Cao Y., Lu J., Chen S., Lenahan C., Zhang J.H., Shao A., Zhang J. (2020). Programmed Cell Deaths and Potential Crosstalk With Blood-Brain Barrier Dysfunction After Hemorrhagic Stroke. Front. Cell Neurosci..

[78] Blecharz-Lang K.G., Wagner J., Fries A., Nieminen-Kelha M., Rosner J., Schneider U.C., Vajkoczy P. (2018). Interleukin 6-Mediated Endothelial Barrier Disturbances Can Be Attenuated by Blockade of the IL6 Receptor Expressed in Brain Microvascular Endothelial Cells. Transl. Stroke Res..

[79] Akamatsu Y., Pagan V.A., Hanafy K.A. (2020). The role of TLR4 and HO-1 in neuroinflammation after subarachnoid hemorrhage. J. Neurosci. Res..

[80] Gallia G.L., Tamargo R.J. (2006). Leukocyte-endothelial cell interactions in chronic vasospasm after subarachnoid hemorrhage. Neurol. Res..

[81] Chen J., Wang L., Xu H., Xing L., Zhuang Z., Zheng Y., Li X., Wang C., Chen S., Guo Z. (2020). Meningeal lymphatics clear erythrocytes that arise from subarachnoid hemorrhage. Nat. Commun..

[82] Pradilla G., Chaichana K.L., Hoang S., Huang J., Tamargo R.J. (2010). Inflammation and cerebral vasospasm after subarachnoid hemorrhage. Neurosurg. Clin. N. Am..

[83] Schneider U.C., Schiffler J., Hakiy N., Horn P., Vajkoczy P. (2012). Functional analysis of Pro-inflammatory properties within the cerebrospinal fluid after subarachnoid hemorrhage in vivo and in vitro. J. Neuroinflammation.

[84] Ahn S.H., Savarraj J.P.J., Parsha K., Hergenroeder G.W., Chang T.R., Kim D.H., Kitagawa R.S., Blackburn S.L., Choi H.A. (2019). Inflammation in delayed ischemia and functional outcomes after subarachnoid hemorrhage. J. Neuroinflammation.

[85] Mitsui K., Ikedo T., Kamio Y., Furukawa H., Lawton M.T., Hashimoto T. (2020). TLR4 (Toll-Like Receptor 4) Mediates the Development of Intracranial Aneurysm Rupture. Hypertension.

[86] Liu L., Zhang Q., Xiong X.Y., Gong Q.W., Liao M.F., Yang Q.W. (2018). TLR4 gene polymorphisms rs11536889 is associated with intra cranial aneurysm susceptibility. J. Clin. Neurosci..

[87] Tang A.T., Choi J.P., Kotzin J.J., Yang Y., Hong C.C., Hobson N., Girard R., Zeineddine H.A., Lightle R., Moore T. (2017). Endothelial TLR4 and the microbiome drive cerebral cavernous malformations. Nature.

[88] Hemmer S., Senger S., Griessenauer C.J., Simgen A., Oertel J., Geisel J., Hendrix P. (2022). Admission serum high mobility group box 1 (HMGB1) protein predicts delayed cerebral ischemia following aneurysmal subarachnoid hemorrhage. Neurosurg. Rev..

[89] Harris H.E., Andersson U., Pisetsky D.S. (2012). HMGB1: A multifunctional alarmin driving autoimmune and inflammatory disease. Nat. Rev. Rheumatol..

[90] Chaudhry S.R., Guresir A., Stoffel-Wagner B., Fimmers R., Kinfe T.M., Dietrich D., Lamprecht A., Vatter H., Guresir E., Muhammad S. (2018). Systemic High-Mobility Group Box-1: A Novel Predictive Biomarker for Cerebral Vasospasm in Aneurysmal Subarachnoid Hemorrhage. Crit. Care Med..

[91] Zhao X.D., Mao H.Y., Lv J., Lu X.J. (2016). Expression of high-mobility group box-1 (HMGB1) in the basilar artery after experimental subarachnoid hemorrhage. J. Clin. Neurosci..

[92] Zhu X.D., Chen J.S., Zhou F., Liu Q.C., Chen G., Zhang J.M. (2012). Relationship between plasma high mobility group box-1 protein levels and clinical outcomes of aneurysmal subarachnoid hemorrhage. J. Neuroinflammation.

[93] Ieong C., Sun H., Wang Q., Ma J. (2018). Glycyrrhizin suppresses the expressions of HMGB1 and ameliorates inflammative effect after acute subarachnoid hemorrhage in rat model. J. Clin. Neurosci..

[94] Haruma J., Teshigawara K., Hishikawa T., Wang D., Liu K., Wake H., Mori S., Takahashi H.K., Sugiu K., Date I. (2016). Anti-high mobility group box-1 (HMGB1) antibody attenuates delayed cerebral vasospasm and brain injury after subarachnoid hemorrhage in rats. Sci. Rep..

[95] Gram M., Sveinsdottir S., Ruscher K., Hansson S.R., Cinthio M., Akerstrom B., Ley D. (2013). Hemoglobin induces inflammation after preterm intraventricular hemorrhage by methemoglobin formation. J. Neuroinflammation.

[96] Wang K.C., Tang S.C., Lee J.E., Lai D.M., Huang S.J., Hsieh S.T., Jeng J.S., Tu Y.K. (2014). Prognostic value of intrathecal heme oxygenase-1 concentration in patients with Fisher Grade III aneurysmal subarachnoid hemorrhage. J. Neurosurg..

[97] Frase S., Steimer M., Selzner L., Kaiser S., Foit N.A., Niesen W.D., Schallner N. (2022). Temporal Expression Pattern of Hemoxygenase-1 Expression and Its Association with Vasospasm and Delayed Cerebral Ischemia After Aneurysmal Subarachnoid Hemorrhage. Neurocrit. Care.

[98] Bozza M.T., Jeney V. (2020). Pro-inflammatory Actions of Heme and Other Hemoglobin-Derived DAMPs. Front. Immunol..

[99] Figueiredo R.T., Fernandez P.L., Mourao-Sa D.S., Porto B.N., Dutra F.F., Alves L.S., Oliveira M.F., Oliveira P.L., Graca-Souza A.V., Bozza M.T. (2007). Characterization of heme as activator of Toll-like receptor 4. J. Biol. Chem..

[100] Becher B., Spath S., Goverman J. (2017). Cytokine networks in neuroinflammation. Nat. Rev. Immunol..

[101] Ridwan S., Grote A., Simon M. (2021). Interleukin 6 in cerebrospinal fluid is a biomarker for delayed cerebral ischemia (DCI) related infarctions after aneurysmal subarachnoid hemorrhage. Sci. Rep..

[102] Lenski M., Huge V., Briegel J., Tonn J.C., Schichor C., Thon N. (2017). Interleukin 6 in the Cerebrospinal Fluid as a Biomarker for Onset of Vasospasm and Ventriculitis After Severe Subarachnoid Hemorrhage. World Neurosurg..

[103] Wu W., Guan Y., Zhao G., Fu X.J., Guo T.Z., Liu Y.T., Ren X.L., Wang W., Liu H.R., Li Y.Q. (2016). Elevated IL-6 and TNF-alpha Levels in Cerebrospinal Fluid of Subarachnoid Hemorrhage Patients. Mol. Neurobiol..

[104] Hanafy K.A., Stuart R.M., Khandji A.G., Connolly E.S., Badjatia N., Mayer S.A., Schindler C. (2010). Relationship between brain interstitial fluid tumor necrosis factor-alpha and cerebral vasospasm after aneurysmal subarachnoid hemorrhage. J. Clin. Neurosci..

[105] Galea J., Ogungbenro K., Hulme S., Patel H., Scarth S., Hoadley M., Illingworth K., McMahon C.J., Tzerakis N., King A.T. (2018). Reduction of inflammation after administration of interleukin-1 receptor antagonist following aneurysmal subarachnoid hemorrhage: Results of the Subcutaneous Interleukin-1Ra in SAH (SCIL-SAH) study. J. Neurosurg..

[106] Singh N., Hopkins S.J., Hulme S., Galea J.P., Hoadley M., Vail A., Hutchinson P.J., Grainger S., Rothwell N.J., King A.T. (2014). The effect of intravenous interleukin-1 receptor antagonist on inflammatory mediators in cerebrospinal fluid after subarachnoid haemorrhage: A phase II randomised controlled trial. J. Neuroinflammation.

[107] Sarrafzadeh A., Schlenk F., Gericke C., Vajkoczy P. (2010). Relevance of cerebral interleukin-6 after aneurysmal subarachnoid hemorrhage. Neurocrit. Care.

[108] Smith C.J., Hulme S., Vail A., Heal C., Parry-Jones A.R., Scarth S., Hopkins K., Hoadley M., Allan S.M., Rothwell N.J. (2018). SCIL-STROKE (Subcutaneous Interleukin-1 Receptor Antagonist in Ischemic Stroke): A Randomized Controlled Phase 2 Trial. Stroke.

[109] Sokol B., Wasik N., Jankowski R., Holysz M., Wieckowska B., Jagodzinski P. (2016). Soluble Toll-Like Receptors 2 and 4 in Cerebrospinal Fluid of Patients with Acute Hydrocephalus following Aneurysmal Subarachnoid Haemorrhage. PLoS ONE.

[110] Matsunaga N., Tsuchimori N., Matsumoto T., Ii M. (2011). TAK-242 (resatorvid), a small-molecule inhibitor of Toll-like receptor (TLR) 4 signaling, binds selectively to TLR4 and interferes with interactions between TLR4 and its adaptor molecules. Mol. Pharmacol..

[111] Okada T., Kawakita F., Nishikawa H., Nakano F., Liu L., Suzuki H. (2019). Selective Toll-Like Receptor 4 Antagonists Prevent Acute Blood-Brain Barrier Disruption After Subarachnoid Hemorrhage in Mice. Mol. Neurobiol..

[112] Okada T., Lei L., Nishikawa H., Nakano F., Nakatsuka Y., Suzuki H. (2020). TAK-242, Toll-Like Receptor 4 Antagonist, Attenuates Brain Edema in Subarachnoid Hemorrhage Mice. Acta Neurochir. Suppl..

[113] Liu F.Y., Cai J., Wang C., Ruan W., Guan G.P., Pan H.Z., Li J.R., Qian C., Chen J.S., Wang L. (2018). Fluoxetine attenuates neuroinflammation in early brain injury after subarachnoid hemorrhage: A possible role for the regulation of TLR4/MyD88/NF-kappaB signaling pathway. J. Neuroinflammation.

[114] Rahimifard M., Maqbool F., Moeini-Nodeh S., Niaz K., Abdollahi M., Braidy N., Nabavi S.M., Nabavi S.F. (2017). Targeting the TLR4 signaling pathway by polyphenols: A novel therapeutic strategy for neuroinflammation. Ageing Res. Rev..

[115] Lee J.W., Ahn J.Y., Hasegawa S., Cha B.Y., Yonezawa T., Nagai K., Seo H.J., Jeon W.B., Woo J.T. (2009). Inhibitory effect of luteolin on osteoclast differentiation and function. Cytotechnology.

[116] Yang Y., Tan X., Xu J., Wang T., Liang T., Xu X., Ma C., Xu Z., Wang W., Li H. (2020). Luteolin alleviates neuroinflammation via downregulating the TLR4/TRAF6/NF-kappaB pathway after intracerebral hemorrhage. Biomed Pharmacother..

[117] Park S.J., Song H.Y., Youn H.S. (2009). Suppression of the TRIF-dependent signaling pathway of toll-like receptors by isoliquiritigenin in RAW264.7 macrophages. Mol. Cells.

[118] Zhu H.T., Bian C., Yuan J.C., Chu W.H., Xiang X., Chen F., Wang C.S., Feng H., Lin J.K. (2014). Curcumin attenuates acute inflammatory injury by inhibiting the TLR4/MyD88/NF-kappaB signaling pathway in experimental traumatic brain injury. J. Neuroinflammation.

[119] Sun X.G., Ma Q., Jing G., Wang G.Q., Hao X.D., Wang L. (2017). Increased levels of soluble triggering receptor expressed on myeloid cells-1 in cerebrospinal fluid of subarachnoid hemorrhage patients. J. Clin. Neurosci..

[120] Hirsch Y., Geraghty J.R., Katz E.A., Testai F.D. (2021). Inflammasome Caspase-1 Activity is Elevated in Cerebrospinal Fluid After Aneurysmal Subarachnoid Hemorrhage and Predicts Functional Outcome. Neurocrit. Care.

[121] Wu Q., Wang X.L., Yu Q., Pan H., Zhang X.S., Zhang Q.R., Wang H.D., Zhang X. (2016). Inflammasome Proteins in Cerebrospinal Fluid of Patients with Subarachnoid Hemorrhage are Biomarkers of Early Brain Injury and Functional Outcome. World Neurosurg..

[122] Coll R.C., Hill J.R., Day C.J., Zamoshnikova A., Boucher D., Massey N.L., Chitty J.L., Fraser J.A., Jennings M.P., Robertson A.A.B. (2019). MCC950 directly targets the NLRP3 ATP-hydrolysis motif for inflammasome inhibition. Nat. Chem. Biol..

[123] Ismael S., Zhao L., Nasoohi S., Ishrat T. (2018). Inhibition of the NLRP3-inflammasome as a potential approach for neuroprotection after stroke. Sci. Rep..

[124] Wang H., Chen H., Jin J., Liu Q., Zhong D., Li G. (2020). Inhibition of the NLRP3 inflammasome reduces brain edema and regulates athe distribution of aquaporin-4 after cerebral ischaemia-reperfusion. Life Sci..

